# Exergetic and Economic Evaluation of a Transcritical Heat-Driven Compression Refrigeration System with CO_2_ as the Working Fluid under Hot Climatic Conditions

**DOI:** 10.3390/e21121164

**Published:** 2019-11-28

**Authors:** Jing Luo, Tatiana Morosuk, George Tsatsaronis, Bourhan Tashtoush

**Affiliations:** 1 Institute for Energy Engineering, Technische Universität Berlin, Marchstr. 18, 10587 Berlin, Germany; tetyana.morozyuk@tu-berlin.de (T.M.); georgios.tsatsaronis@tu-berlin.de (G.T.); 2 Mechanical Engineering Department, Jordan University of Science and Technology, Ar Ramtha 3030, Jordan; bourhan@just.edu.jo

**Keywords:** transcritical heat-driven refrigeration machine, carbon dioxide, optimization, exergy analysis, economic analysis

## Abstract

The purpose of this research is to evaluate a transcritical heat-driven compression refrigeration machine with CO_2_ as the working fluid from thermodynamic and economic viewpoints. Particular attention was paid to air-conditioning applications under hot climatic conditions. The system was simulated by Aspen HYSYS^®^ (AspenTech, Bedford, MA, USA) and optimized by automation based on a genetic algorithm for achieving the highest exergetic efficiency. In the case of producing only refrigeration, the scenario with the ambient temperature of 35 °C and the evaporation temperature of 5 °C showed the best performance with 4.7% exergetic efficiency, while the exergetic efficiency can be improved to 22% by operating the system at the ambient temperature of 45 °C and the evaporation temperature of 5 °C if the available heating capacity within the gas cooler is utilized (cogeneration operation conditions). Besides, an economic analysis based on the total revenue requirement method was given in detail.

## 1. Introduction

Refrigeration systems with different configurations, thermodynamic cycles, and working fluids are used for maintaining the temperature of an object below the ambient temperature. They are also considered as one of the essential parts of various industries/applications, e.g., food preservation, air conditioning, chemical industries, and biotechnologies. For the areas without a secure power supply, heat-driven compression refrigeration machine is considered as one of the most promising technologies since it has several unique advantages, for example, high efficiency, simple design, as well as the opportunity of utilizing an inexpensive low-grade energy source.

The first heat-driven compression refrigeration machine was proposed by Chistiakov and Plotnikov (USSR) in the year of 1952 and reported in detail by Rosenfeld and Tkachev [1]. The idea was to “drive” a direct Organic Rankine Cycle (ORC) by the thermal energy, while the shaft work generated from the ORC was further used to operate a vapor-compression refrigeration cycle (VCRC). Two subcycles were combined by a mutual condenser. In terms of the working fluid, any of the working fluids—in general, either one-component or mixtures that are used for refrigeration cycles—could be applied [2,3], and the same working fluid was used for both subcycles. During some period, the heat-driven compression refrigeration machines were out of the research interest. Recently, the interest in these machines has been renewed.

Aphornratana and Sriveerakul [4] proposed a modified combined cycle of an ORC and a VCRC, which integrated the expander and compressor into a free-piston unit. R22 and R134a were examined as the working fluids for the overall system. The results showed that the system achieved better coefficient of performance (*COP*) with R22. A prototype of an ORC–VCRC system with 5 kW refrigeration capacity was tested in the laboratory and reported by Wang et al. [5]. R-245fa and R-134a were selected as the working fluids for the power and the refrigeration sides, respectively. Since two different working fluids were applied, two separate condensers in this experiment were used rather than a mutual condenser, and the subcycles were only connected via the shaft work between the expander of the power cycle and the compressor of the refrigeration cycle. The same research group also investigated and compared three design configurations of the heat-driven refrigeration cycle [6]. The best cycle (with subcooling and cooling recuperation) showed a 22% improvement in terms of the *COP* to the base case. In this research, the same working fluid (R-245fa) for both the power cycle and refrigeration cycle was used. Thus, two subcycles were able to be combined through a mutual condenser. Bu et al. [7] focused on the discovery of the most suitable working fluid for an ORC–VCRC ice-maker driven by solar energy. Four working fluids—R123, R-245fa, R-600a, and R600—were examined regarding the overall energy efficiency. The results revealed that R123 had the best performance under the defined operating conditions. 

Four primary hydrocarbon working fluids were analyzed for an ORC–VCRC system for achieving better environmental performance (Li et al. [8]). With the assumption that the low-grade thermal energy will be supplied, the conclusion was that R600 was the most promising working fluid with the overall *COP* of 0.47. Similar research [9,10] was carried out to evaluate the performance of four working fluids with low Global Warming Potential (GWP) for a low-temperature heat-driven ORC–VCRC system. R-1336mzz(Z) and R-1233zd(E) were applied for the ORC, while R1234yf and R1234ze(E) were considered for the refrigeration cycle. The system performance for four different combinations of working fluids was thoroughly conducted. The results showed that the choice of the working fluid for the VCRC had only a slight effect on the overall system efficiency. In addition, the combination of R1336mzz(Z) for the power cycle and R1234ze(E) for the refrigeration cycle resulted in the highest energetic efficiency. Kim and Perez-Blanco [11] discussed a cogeneration system (producing power and refrigeration) by applying the heat-driven refrigeration machine. A limiting case was mentioned as solely producing cold without net electricity by controlling the flow division ratio. They concluded that the system exergetic efficiency was proportional to the refrigeration capacity with a fixed source temperature and a given mass flow rate of the ORC. Nasir and Kim [12] investigated seven working fluids with forty-nine combined options for an ORC–VCRC system driven by low-grade heat. The ambient temperature was assumed between 30–40 °C, and the system was designed for air conditioning purposes with a room temperature of 15 °C. The best combination among the forty-nine options was R-134a and isobutane, with *COP* of 0.22.

In the publications mentioned above, one can conclude that selecting the suitable working fluid is crucial for designing and operating the heat-driven ORC–VCRC systems. The selection of the working fluid directly influences the thermodynamic performance of the system as well as the environmental performance. Besides, the safety, the reliability, and the cost of the working fluid should also be taken into consideration. CO_2_ (R744) as a natural working fluid is getting more and more attention and has been extensively researched since it is nontoxic, nonflammable, inexpensive, and environmentally benign. For example, Lorentzen and Pettersen [13], as well as Cavallini and Zilio [14], discussed deeply how promising CO_2_ would be as a natural working fluid in the future. The low critical temperature (31.1 °C) and the high critical pressure (73.8 bar) of CO_2_ in conjunction with its thermodynamic properties (slightly above critical point and near saturation lines) create a high potential for improving the thermodynamic and economic effectiveness of the refrigeration systems. 

However, the research of using CO_2_ as a refrigerant focused mainly on the transcritical VCRC. For example, Rozhentsev and Wang [15] discussed the thermodynamic efficiency of the heat regeneration within the transcritical VCRC, while Shiferaw at al. [16] evaluated the economic potential for transcritical VCRC systems, and Fazelpour and Morosuk [17] proposed two optimal configurations of the transcritical VCRC with economizer from the exergoeconomic analysis point of view. The idea of implementing ejector technologies to a transcritical cascade refrigeration cycle was also reported [18,19]. The information reported for the transcritical heat-driven refrigeration cycle considering CO_2_ as the working fluid was minimal. In general, compared to conventional vapor-compression refrigeration machines, it is advantageous to employ thermally-driven vapor compression refrigeration machines to ensure the stable refrigeration capacity (for food and vaccine preservation, and/or for air conditioning) for the areas without a secure power supply. Besides, driving the vapor-compression refrigeration machines by heat offers more system flexibility as it is possible to integrate the system into other systems by utilizing any kinds of heat sources, and the system can produce not only refrigeration capacity but also power and heating capacities based on the local requirements. Using CO_2_ as the working fluid for a heat-driven VCRC provides additional potentials for reducing the size of the system, for improving the system efficiency and for reducing the system cost as well. This is appealing for waste heat recovery applications to improve the system efficiency, for ship and automotive applications due to the limited space, and for offices, hotels, and other buildings where refrigeration, power, and heating capacities are needed simultaneously.

The performance of a waste heat-driven vapor compression refrigeration machine using CO_2_ as the working fluid has been discussed by authors [20]. The system was designed to utilize the low-grade waste heat, and four scenarios with various evaporation temperatures were evaluated and compared for storage of a wide range of food products and air conditioning applications. This work aimed to pay special attention to air conditioning applications for countries or regions having hot climates (for example, the Middle East, India, and South China) since these countries/regions are developing substantially and with massive populations, which leads to considerable energy consumption for air conditioning purpose.

## 2. System Description

**Figure 1:**
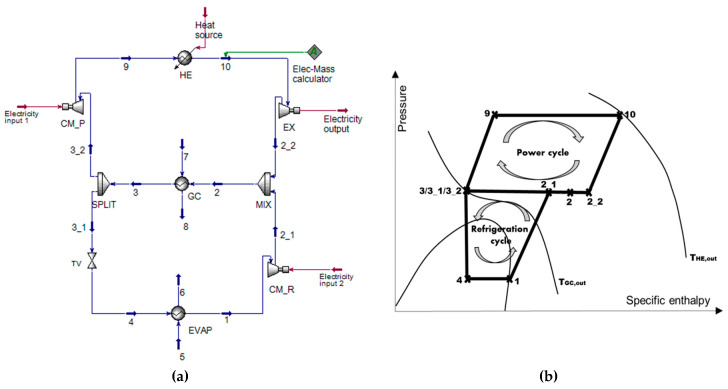
(**a**) Schematic and (**b**) thermodynamic cycle of a transcritical heat-driven compression refrigeration machine with R744.

Figure 1 presents the schematic of a transcritical heat-driven compression refrigeration system with R744 inspired by Chistiakov and Plotnikov [1]. The system coupled a closed power cycle (Brayton cycle) with a transcritical refrigeration cycle. It consists of nine components: an evaporator (EVAP), a compressor for refrigeration cycle (CM_R), a mixer (MIX), a gas cooler (GC), a splitter (SPLIT), a throttling valve (TV), a compressor for power cycle (CM_P) as well as a heater (HE) and an expander (EX).

The features of the new system are as follows:

- R744 is the only working fluid for both subsystems.
- The power cycle operates entirely in the supercritical region, and part of the refrigeration cycle is above the critical point.
- The shafts of the expander and two compressors are directly connected.
- The refrigeration capacity of the evaporator is the main product.
- The net shaft work (can be further converted to electricity), which is the difference between the power generation of the expander and the total power consumption of two compressors, can be produced as the second product of the system.
- The available heating capacity within the gas cooler can be considered as the third product depending on the local requirements.
- Any kinds of heat sources, in general, can be used for driving the system, for example, solar thermal energy, geothermal heat, heat from biomass and waste heat from chemical plants and internal combustion engines. The low-medium grade waste heat was focused in this study.

Besides,

- the machine is used for air conditioning purposes (*T_EVAP_* = 5 and 15 °C [21]), and
- hot climatic operation conditions were assumed (*T*_0_ = 35 and 45 °C [17]).

The following assumptions were made for the simulations:

- The refrigeration capacity is 100 kW.
- The shaft work generated from the expander is merely sufficient to power both compressors, ${\dot{W}}_{\mathrm{net}}=0\;\mathrm{kW}$.
- The temperature of cooling water (stream 7) is equal to the assumed environmental temperature, *T_7_* = *T*_0_.
- The temperature of “heat source” (HS) for the heater is always 20 K higher than the turbine inlet temperature (TIT): *T_HS_* = *T_TIT_* + 20 K. Since the system in this work is considered to be driven by waste heat (for example, waste heat from flue gases), a gas–gas heat exchanger is assumed.
- The outlet stream from the evaporator (stream 1) is saturated vapor (since it has been proved that the effect of the superheating process within the evaporator for transcritical refrigeration machines can be neglected [15]).
- The isentropic efficiency of both compressors, CM_P and CM_R (turbo-compressor), is equal to 0.85 [21].
- The isentropic efficiency of the expander (turbo-expander) is assumed to be equal to 0.9 [21].
- The gas cooler and the evaporator are considered to operate with the pinch temperature difference of 5 K.
- The simulation is completed under steady-state conditions. The pressure drops in pipes, heat exchangers, as well as within the mixer and the splitter are neglected.

## 3. Methods

The simulations were carried out by Aspen HYSYS^®^ Software (AspenTech, Bedford, MA, USA). Moreover, the exergy-based method was applied for optimizing, comparing, and investigating the system under various operation conditions. The Span–Wagner equation of state was selected for calculating the thermodynamic properties of CO_2_ since it is one of the most accurate models to predict CO_2_ behaviors in a wide range of temperature and pressure, including a high temperature, at high pressure and in the vicinity of its critical point [22]. To conduct the exergy-based method, the reference temperature *T*_0_ varies when the assumption of the ambient temperature varies (*T*_0_ = 35/45 °C), while the reference pressure *p*_0_ in this study keeps constant (*p*_0_ = 1.013 bar).

### 3.1. Optimization

The system optimization was first carried out for achieving the highest exergetic efficiency of the system operating under different conditions. Aspen HYSYS was connected with the programming language, Python, through a binary-interface, component object model (COM), which allows the communication between these two programs. A genetic algorithm (GA) as a metaheuristic optimization technique was selected to conduct the optimization. The algorithm is inspired by Charles Darwin’s theory, which describes the process of natural evolution to solve complex optimization problems. The fittest individual will be finally generated and selected after several generations through selection, crossover, and mutation procedures [23]. In this study, the objective function was defined to maximize the exergetic efficiency of the overall system. The population size was set at 100, the uniform crossover was applied, and the mutation rate was tuned as 0.3. The optimization procedure terminates after ten iterations, and the constraints of the design variables are listed in Table 1.

The expander inlet operation conditions are *T*_10_ and *p*_10_. The pressure ratio within the compressors is PRc. The outlet stream of R744 from the GC is with *T*_3_ and *p*_3_. The maximum pressure for operating the supercritical CO_2_ power cycle is assumed to be 200 bar [21,26,27] because higher operating pressure will lead to thicker walls and more expensive materials, which increases the cost of the overall system.

### 3.2. Analysis

The comparison of the system with different ambient temperatures and evaporation temperatures was then implemented from energetic, exergetic, and economic viewpoints. The equations of energetic and exergetic evaluations were given from different perspectives by considering the system as the refrigeration system, cogeneration system, and trigeneration system.

#### 3.2.1. Energetic Analysis

The energetic efficiency of the overall system can be defined as follows:

• for trigeneration (heat, refrigeration, and power):

(1) $$COP_{Overall}=\left({\dot{Q}}_{GC}+{\dot{W}}_{net}\right)/{\dot{Q}}_{HE},$$

• for cogeneration (refrigeration and heat):

(2) $$COP_{Overall}={\dot{Q}}_{GC}/{\dot{Q}}_{HE},$$

• for cogeneration (refrigeration and power):

(3) $$COP_{Overall}=\left({\dot{Q}}_{EVAP}+{\dot{W}}_{net}\right)/{\dot{Q}}_{HE},$$

• for only refrigeration:

(4) $$COP_{Overall}={\dot{Q}}_{EVAP}/{\dot{Q}}_{HE}.$$

While, for two subsystems, the expressions of the energetic efficiency are as follows:

The closed power cycle, $\eta_{P}$

• for trigeneration (heat, refrigeration, and power) or cogeneration (refrigeration and heat):

(5) $$\eta_{P}=\left({\dot{W}}_{EX}-{\dot{W}}_{CM\_P}+{\dot{Q}}_{GC,P}\right)/{\dot{Q}}_{HE},$$

• for cogeneration (refrigeration and power) or only refrigeration:

(6) $$\eta_{P}=\left({\dot{W}}_{EX}-{\dot{W}}_{CM\_P}\right)/{\dot{Q}}_{HE},\;$$

the refrigeration cycle, *COP*

• for trigeneration (heat, refrigeration, and power) or cogeneration (refrigeration and heat):

(7) $$COP={\dot{Q}}_{GC,R}/{\dot{W}}_{CM\_R},$$

• for cogeneration (refrigeration and power) or only refrigeration:

(8) $$COP={\dot{Q}}_{EVAP}/{\dot{W}}_{CM\_R}.$$

Here, ${\dot{Q}}_{EVAP}$ is the desired refrigeration capacity, ${\dot{W}}_{net}$ is the net power output, ${\dot{Q}}_{GC}$ is the available heating capacity, and ${\dot{Q}}_{HE}$ is the heat absorbed from the heat sources. ${\dot{Q}}_{GC,P}$ and ${\dot{Q}}_{GC,R}$ are the heat capacities contributed by the power cycle and the refrigeration cycle, respectively, if the system is treated as two subsystems.

The ${\dot{W}}_{net}$ is expressed as

(9) $${\dot{W}}_{net}={\dot{W}}_{EX}-{\dot{W}}_{CM\_P}-{\dot{W}}_{CM\_R}.$$

${\dot{Q}}_{GC,P}$ and ${\dot{Q}}_{GC,R}$ are proportional to the mass flow rate ratios of the power cycle mass flow rate and refrigeration cycle mass flow rate to the overall mass flow rate, respectively. Additionally, the sum of ${\dot{Q}}_{GC,P}$ and ${\dot{Q}}_{GC,R}$ should equal to the total heat capacity within the GC, ${\dot{Q}}_{GC}$:

(10) $${\dot{Q}}_{GC,P}=\frac{\dot{m_{P}}}{\left(\dot{m_{P}}+\dot{m_{R}}\right)}*{\dot{Q}}_{GC},$$

(11) $${\dot{Q}}_{GC,R}=\frac{\dot{m_{R}}}{\left(\dot{m_{P}}+\dot{m_{R}}\right)}*{\dot{Q}}_{GC},$$

(12) $${\dot{Q}}_{GC,P}+{\dot{Q}}_{GC,R}={\dot{Q}}_{GC}.\;$$

#### 3.2.2. Exergetic Analysis

In addition to energy analysis, the exergetic evaluation was conducted based on the exergy of fuel/exergy of product approach (for the kth component and the overall system) to identify the location and the magnitude of the irreversibilities [29]. The exergetic efficiency ε is expressed as the ration of exergy of product (${\dot{E}}_{P}$) and exergy of fuel (${\dot{E}}_{F}$):

(13) $$\varepsilon ={\dot{E}}_{P}/{\dot{E}}_{F}.$$

Neglecting the variations of the potential exergy, the kinetic exergy, as well as the chemical exergy, only physical exergy was considered in this work. However, the physical exergy of the material stream needs to be split into the thermal (${\dot{E}}^{T}$) and mechanical (${\dot{E}}^{M}$) parts since several components operate below and cross the ambient temperature [30]:

(14) $${\dot{E}}^{PH}={\dot{E}}^{T}+{\dot{E}}^{M}.$$

For the kth component, the value difference between the exergy of fuel and the exergy of the product is the exergy destruction, which indicates the irreversibilities within the component:

(15) $${\dot{E}}_{F,k}-{\dot{E}}_{P,k}={\dot{E}}_{D,k},$$

while, for the overall system, the difference between the fuel and the product is not only the exergy destruction but also the exergy loss (${\dot{\;E}}_{L,tot}$), which is defined as the exergy transferred into the environment [29]:

(16) $${\dot{E}}_{F,tot}-{\dot{E}}_{P,tot}={\dot{E}}_{D,tot\;}+{\dot{\;E}}_{L,tot}.$$

The definitions of the fuel, the product, the destruction, and the loss for each component, as well as for the overall system, are given in Table 2. The fuel and the product for the mixer were not defined since the mixer was considered as a dissipative component [29]. Moreover, for the overall system, various product definitions are given regarding the total number of products that have been considered.

#### 3.2.3. Economic Analysis

To analyze the system from an economic viewpoint, the total revenue requirement (TRR) method was applied [29]. For conducting the TRR, the total capital investment (TCI) of the system was first estimated based on the purchased equipment cost (PEC) of each component, then the economic, financial, operating, and market input parameters were determined for the detailed cost calculation. Finally, the geometrically increasing series of expenditures will be levelized into a financially equivalent constant quantity (annuity). The final equation for calculating the levelized total revenue requirement is written as

(17) $$TRR_{L}=CC_{L}+FC_{L}+OMC_{L},\;$$

where $CC_{L}$ stands for levelized carrying charges, $FC_{L}$ is the levelized fuel cost, and $OMC_{L}$ is for levelized operating and maintenance costs.

In addition, the $CC_{L}$ is calculated as

(18) $$CC_{L}=TCI*CRF,\;$$

where CRF is the capital recovery factor and can be given by

(19) $$CRF=\frac{i_{eff}{\left(1+i_{eff}\right)}^{n}}{{\left(1+i_{eff}\right)}^{n}-1},$$

$i_{eff}$ and *n* are the effective interest rate and the economic lifetime of the power plant, respectively.

$FC_{L}$ is determined by the fuel cost at the beginning of the first year $FC_{0}$ and the constant escalation levelization factor (*CELF*):

(20) $$FC_{L}=FC_{0}*CELF=FC_{0}*\frac{k_{FC}\left(1-k_{FC}^{n}\right)}{1-k_{FC}}*CRF,$$

with

(21) $$k_{FC}=\frac{1+r_{FC}}{1+i_{eff}}.$$

The same procedure was applied for $OMC_{L}$:

(22) $$OMC_{L}=OMC_{0}*CELF=OMC_{0}*\frac{k_{OMC}\left(1-k_{OMC}^{n}\right)}{1-k_{OMC}}*CRF,$$

with

(23) $$k_{OMC}=\frac{1+r_{OMC}}{1+i_{eff}},$$

where $r_{FC}$ and $r_{OMC}$ stand for the average inflation rate of the fuel cost and the operating and maintenance cost, respectively.

All the assumptions made for the economic analysis are summarized in Table 3. The fuel cost was assumed to be free of charge since the heat source is unknown.

The key and the most challenging part of an economic evaluation is to estimate the *PEC* of each component, especially for the new and uncommercialized technology. Therefore, in the following sections, the procedures applied for estimating the *PEC* for all the components of the system are explained in detail.

• HE and GC

Since the HE and GC were expected to work at high-temperature and high-pressure, the printed circuit heat exchanger (PCHE) technology was selected to fulfill the requirements of the closed power cycle rather than a standard shell and tube heat exchanger [16,31]. The PCHE technology is a relatively new technology applied for manufacturing compact heat exchangers by photoetching microchannel technology and a specific solid-state joining process to boost the mechanical integrity and efficiency, technology readiness level, and flexibility of heat exchangers [14,25]. Meanwhile, the capital cost of the overall system was expected to be reduced by replacing the shell and tube heat exchangers by the PCHE. Based on the research of “Heatric” (UK) [32] that has already started to produce PCHEs for supercritical cycle applications, the cost of a PCHE should be estimated by its weight: $Cost_{PCHE}=\;Cost\;per\;unit\;mass_{metal}\;*\;Mass_{PCHE}$, with $Mass_{PCHE}=$ $Density_{metal}*Volume_{metal}$. To calculate the volume of the metal that was used for manufacturing the heat exchanger, the volume fraction of the metal to the heat exchanger per m^3^, $f_{m}$ was needed, $Volume_{metal}=Volume_{PCHE}*f_{m}$. The size of the heat exchanger $Volume_{PCHE}$ can be estimated by the area of the heat exchanger and the information of the typical area per unit volume, $Volume_{PCHE}=\frac{A_{PCHE}}{\mathrm{typical}\;\mathrm{area}\;per\;\mathrm{unit}\;\mathrm{volume}}$. Depending on the operating pressure, the typical area per unit volume for PCHEs is around 1300 m^2^/m^3^ at 100 bar and 650 m^2^/m^3^ at 500 bar [32]. The heat-transfer area of the heat exchanger $A_{PCHE}$, was calculated by the equation *Q = U A T_LMTD_. Q* stands for the transferred heat within the heat exchanger; U is the overall heat-transfer coefficient; and T_LMTD_ is the log mean temperature difference. The assumptions made for estimating the cost of PCHEs are summarized in Table 4.

• Turbomachinery (CM_P and EX)

Since the turbomachinery operating with R744 is not well known for commercial application yet, the costs of the expander and compressor in the closed power cycle were scaled from the available cost information for helium turbomachinery [27]:

(24) $$Temperature\;Proportionality\;Constant=\;3.35+{\left(\frac{TIT}{1000}\right)}^{7.8},$$

(25) $$Pressure\;Proportionality\;Constant\;=\;TIP^{-0.3},$$

(26) $$Power\;Capacity\;Proportionality\;Constant\;=\;P_{G}^{\frac{285}{TIP^{1.7}}+\;0.6},$$

where TIT is the expander inlet temperature in °C, TIP is the expander inlet pressure in bar, and the $P_{G}$ represents the power generated within expander in MW.

• CM_R and EVAP

For the compressor and the evaporator of the transcritical refrigeration cycle, their costs were considered as a function of the capacity. Furthermore, the cost correction factors were also considered regarding materials, design pressure, and design temperature [33]: $C_{E}=C_{B}*{\left(X/X_{B}\right)}^{M}f_{M}f_{P}f_{T}$, where $C_{E}$ is the new equipment cost with capacity X, $C_{B}$ is the known base cost for equipment with capacity $X_{B}$, M is an exponent, and $f_{M},f_{P}\;\mathrm{and}\;f_{T}$ are the correction factors in terms of materials, design pressure, and design temperature, respectively.

Moreover, the power consumption was used as the capacity X for estimating the cost of the compressor, while, for the evaporator, X refers to the heat-transfer area of the heat exchanger. For calculating the heat-transfer area of the evaporator, the overall heat-transfer coefficient was set to 950 W/(m^2^K) [17]. In Table 5, the values used for the cost estimation of the compressor and evaporator are listed.

• *Other* components

For the PECs of the TV and others, the following assumptions were made:

1. For the refrigeration machine with the cooling capacity of 100 kW, the cost of the TV equals to 100 € [17];
2. The costs of the mixer and the splitter are neglected.

Finally, the costs of all components were brought up-to-date using the cost indices [33] from Chemical Engineering (CE) and applied in US$_2017_:

(27) $$Cost\;at\;the\;reference\;year=Original\;cost\;\times \frac{Cost\;index\;for\;the\;reference\;year}{Cost\;index\;for\;the\;original\;year\;cost}.\;$$

## 4. Results and Discussion

### 4.1. Thermodynamic Investigations

In this study, the performance of a transcritical heat-driven refrigeration system with R744 as the working fluid for both subsystems was investigated. Four scenarios focusing on air conditioning applications (*T_EVAP_* = 5 and 15 °C) under hot climatic conditions (*T*_0_ = 35 and 45 °C) were simulated, optimized, and compared. Table 6 demonstrates the exergetic optimization results aiming at the highest exegetic efficiency of the overall system for each scenario.

The results showed that the lowest *T*_10_ but the highest *p*_10_ were selected for each scenario, while the optimal *T*_3_ and *p*_3_ depended on the ambient temperature rather than the evaporation temperature. Hence, the closed power cycle, for various scenarios with the same ambient temperature, was suggested to operate at the same conditions. Only the ${\dot{m}}_{P}$ differed, but the energetic and exergetic efficiencies of the power cycle with the same ambient conditions were consistent. Meanwhile, for the transcritical refrigeration cycle, by changing the evaporation temperature from 5 to 15 °C, the pressure ratio ${\mathrm{PR}}_{R}$ and the ${\dot{m}}_{P}$ varied simultaneously, which affected its energetic and exergetic efficiencies. With the higher evaporation temperature, the higher *COP* of the transcritical refrigeration cycle was achieved as well as the better energy performance of the overall system. While the exergetic efficiencies of the refrigeration cycle and the overall system decreased significantly by increasing the evaporation temperature.

**Figure 2:**
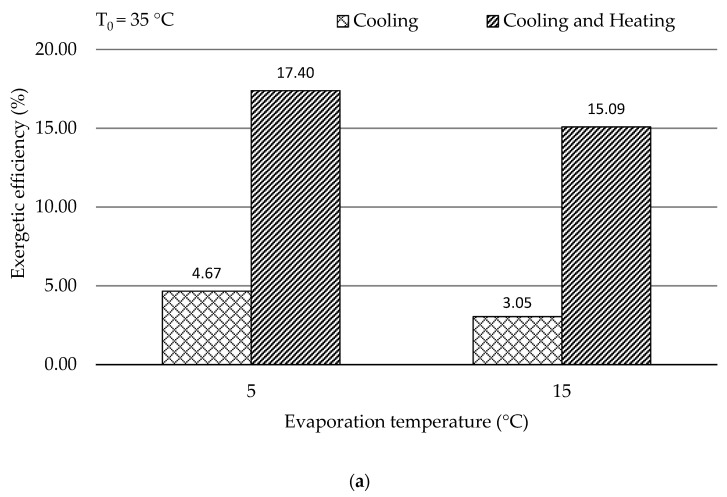

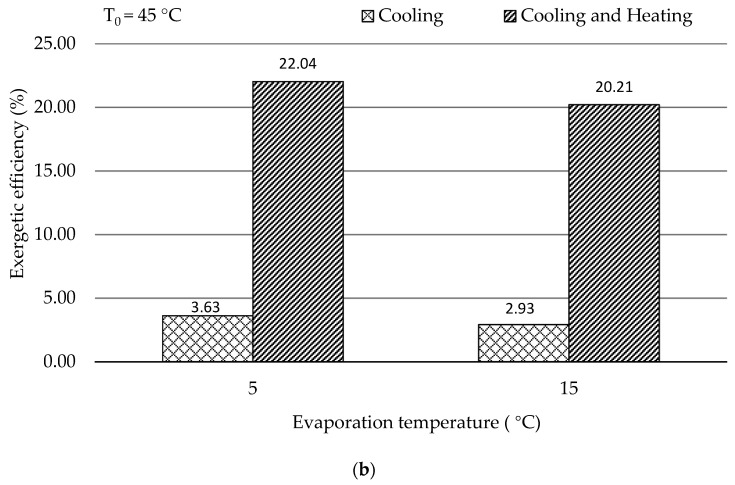
$\varepsilon_{Overall}$ with and without available heating product for different environmental temperatures by varying evaporation temperatures (**a**) *T*_0_ = 35 °C and (**b**) *T*_0_ = 45 °C.

By increasing the ambient temperature, the energetic and the exergetic efficiencies of both subsystems dropped, which resulted in lower performance of the overall system. One can conclude that the system operating with the evaporation temperature of 15 °C (higher evaporation temperature) and the ambient temperature of 35 °C (lower ambient temperature) yielded the best energetic efficiency of 0.39; while the highest exergetic efficiency (4.67%) of the overall system was achieved with lower evaporation temperature (5 °C) and lower ambient temperature (35 °C). In addition, the ratio of the available heating product to the refrigeration product in terms of exergy (${\dot{E}}_{Heating}/{\dot{E}}_{Cooling}$) was notable, particularly at higher ambient temperature. Hence, the overall exergetic efficiencies of the scenarios with and without utilizing the available heat capacity were investigated in Figure 2.

The following conclusions can be made:

- For different ambient operating conditions (ambient temperatures of 35 and 45 °C), the exergetic efficiency of the overall system (with and without available heating product) decreased with increasing the evaporation temperature. Therefore, the system was preferred to operate at a lower evaporation temperature.
- By varying the ambient temperature from 35 to 45 °C, the exergetic efficiency with the consideration of utilizing only refrigeration products reduced, while the efficiency was improved once the heating product was also taken into consideration. It revealed that the ambient temperature had a significant effect on the amount of the available heating capacity, and a large amount of heat, particularly at higher ambient temperature, should be utilized based on the local requirements to improve the performance of the overall system.

**Figure 3:**
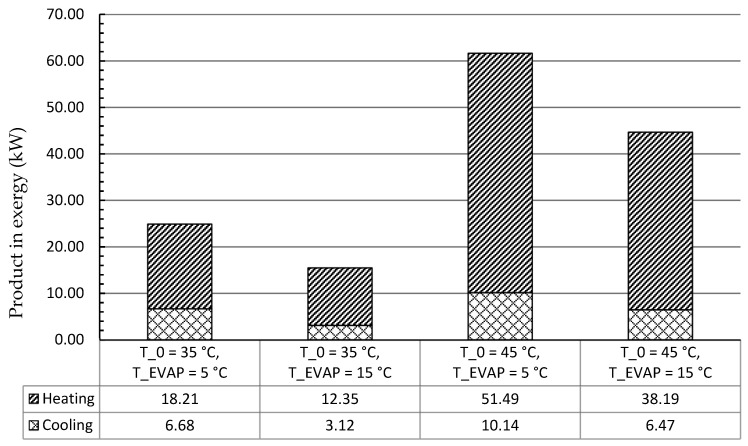
Cooling and heating products in exergy for each scenario (No net power generation).

The products of the system in exergy for each scenario were summarized in Figure 3. Since net power generation was assumed as 0 kW in this study, the products were only considered regarding refrigeration and heat capacities. Around 10 kW of refrigeration capacity and more than 50 kW heat capacity can be produced by the system with the ambient temperature of 45 °C and the evaporation temperature of 5 °C. However, by operating the system at the ambient temperature of 35 °C and the evaporation temperature of 15 °C, only 3.12 kW and 12.35 kW as refrigeration and heat capacities, respectively, were available.

**Figure 4:**
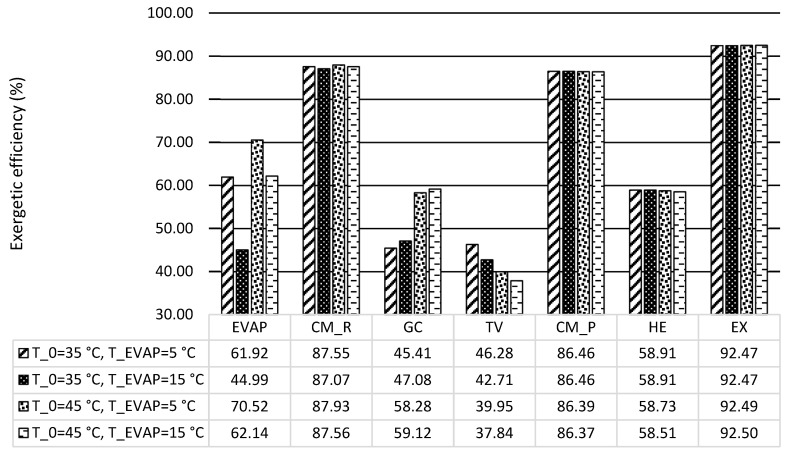
Exergetic efficiency of components for each scenario.

Figure 4 demonstrates the exergetic efficiency of each component for four scenarios. The results showed that the expander and two compressors had the best performance (with exergetic efficiency more than 85%). The heat exchangers (EVAP, GC, and HE) had relatively low efficiency, which was between 45%–70%, while the TV had the lowest efficiency (lower than 45%), which needed to be further considered in order to improve the overall system performance (for example, as it was discussed in [17]). In addition, the variations of exergetic efficiencies within the EVAP, the GC, and the TV were quite notable, since their operating conditions changed considerably by adjusting the evaporation and ambient temperatures.

**Figure 5:**
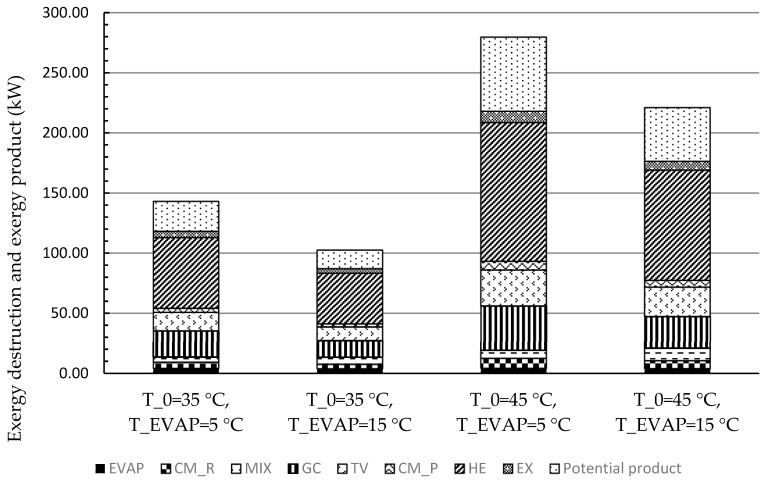
System exergy balance (exergy destruction of each component and potential product of the overall system in exergy) for each scenario.

Figure 5 illustrated the exergy balance of the overall system for each scenario. The part of the exergy associating with components was the exergy destruction within the corresponding component, and the potential product referred to exergy of the overall product (the refrigeration and heat capacities) that can be produced. The sum of the exergy destructions within the components and the potential product of the overall system was the exergy of fuel for the system. It can be noticed that the HE contributed the most to the exergy destruction (more than 40%) since the temperature difference within this component was set as 20 K, which was quite high for the refrigeration application. In addition, the values of the exergy destruction within the GC and the TV were considerable for each scenario. The reason for the high exergy destruction within the GC was due to the high-temperature difference and the high mass flow rate by combining two subsystems. Moreover, the high exergy destruction within the TV can be reduced only by modifying the configurations of the system.

### 4.2. Economic Investigations

**Figure 6:**
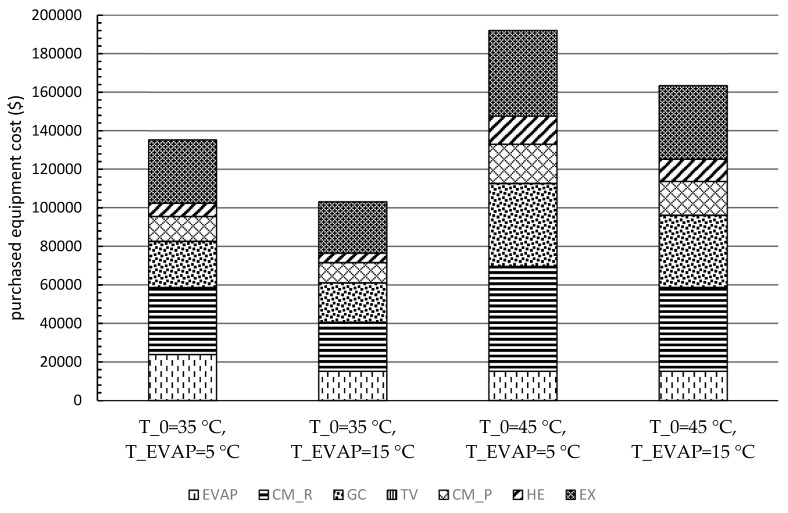
Purchased equipment cost (PEC) of the components for each scenario.

Figure 6 demonstrates the variations of the PECs for each component and the overall system by changing the evaporation and ambient temperatures. The costs of the CM_R, the EX, and the GC dominated the overall PECs for all scenarios, and the sum of their costs reached 74% for the scenario with *T*_0_ = 45 °C and *T_EVAP_* = 5 °C. Moreover, the costs only associated with CM_R and the EX contributed more than half to the total PEC of the overall system for all the operating conditions, while the CM_P had a relatively low cost due to the low power consumption.

The levelized cost of the overall system, which converted the nonuniform annual cost to an equivalent annual constant payment, was summarized in Table 7 for each scenario. In general, the costs of the overall system were significantly influenced by the operating conditions of the transcritical cycle, especially by its optimized pressure ratio. For *T*_0_ = 45 °C with *T_EVAP_* = 5 °C, the system operated with the highest pressure ratio of the transcritical refrigeration cycle, which resulted in the highest cost of the overall system. However, by increasing the evaporation temperature (pressure) and decreasing the operating pressure within the GC, the lowest cost needed to be paid for the scenario of *T*_0_ = 35 °C and *T_EVAP_* = 15 °C. It can be concluded that, from an economic viewpoint, the system operating with lower environmental temperature and higher evaporation temperature was considered with a lower payment, and the pressure ratio of the transcritical refrigeration cycle should be paid attention in the system design phase.

## 5. Conclusions

In this paper, a stand-alone transcritical heat-driven compression refrigeration system using CO_2_ as the working fluid was proposed. Compared to other vapor-compression refrigeration systems, the system, in general, can utilize any kinds of heat sources to produce refrigeration, heating, and power capacities simultaneously. This technology is beneficial to stabilize the refrigeration capacity for the areas without a secure power supply. Moreover, the system using CO_2_—a natural working fluid—as the refrigerant can be driven by renewable energies (for example, solar, geothermal, and biomass energies) and low to medium grade waste heat (for example, waste heat from chemical plants and internal combustion engines), which makes the system attractive from an environmental viewpoint. The low critical temperature (31.1 °C) and the high critical pressure (73.8 bar) of CO_2_ in conjunction with its thermodynamic properties (slightly above critical point and near saturation lines) create a high potential for improving the thermodynamic and economic effectiveness of the refrigeration systems.

In this work, the system for air conditioning purpose was thermodynamically and economically investigated. Special attention was given to countries and regions having hot climates and developing substantially. The refrigeration capacity of the system was assumed as 100 kW, and there was no net power generation. Four scenarios focusing on the air conditioning temperatures under hot climatic conditions were simulated and then optimized, aiming at the highest exergetic efficiency. The optimization results revealed that the five design variables were more sensitive to the ambient temperature rather than the evaporation temperature. With increasing the evaporation temperature, the exergetic efficiency of the overall system decreased. The system performed better, in general, under lower environmental conditions if the refrigeration capacity was considered as the sole product. Since the ratio of the available heat capacity to the refrigeration capacity was significantly boosted by the increment of the environmental temperature, the heat rejection from the GC should be further used for other applications, e.g., the system should operate as a cogeneration/trigeneration system, especially with the higher environmental temperature. With the consideration of utilizing both refrigeration and heat capacities, the system showed the highest exergetic efficiency of 22.04% with *T*_0_ = 45 °C and *T_EVAP_* = 5 °C. The TV and the heat exchangers, especially the GC, had the lowest exergetic efficiency, while the HE, the GC, and the TV were the dominating contributors to the exergy destruction of the overall system. To further improve the performance of the overall system, great attention should be paid to the configurations that can minimize the irreversibilities within the TV and the heat exchangers.

Furthermore, the estimation procedures of the PEC of each component were conducted for four scenarios. The CM_R, the EX, and the GC had the highest PECs for all scenarios. Moreover, the results of the levelized costs based on the TRR method revealed that the annual payment was lower with lower environmental temperature and higher evaporation temperature. However, the cost of the product(s) should be more convincing for comparing the system with various operating conditions, especially by including the heat capacity as the second product for systems under hot climatic conditions. Thus, the exergoeconomic analysis and optimization will be considered as the next step of this research.

## Figures and Tables

**Table 1:** Ranges of design variables. (PRc: pressure ratio within the compressors.).

Design Variable	Range
T10 (°C)	220–500 [24,25]
*p*_10_ (bar)	150–200 [21,26,27]
PRc	1.7–3.0
*T*_3_ (°C)	≥32 [25,28]
*p*_3_ (bar)	≥77 [25,28]

**Table 2:** Exergy of fuel, product, and losses for each component and the overall system.

Component/System	$\mathrm{Exergy}\;\mathrm{of}\;\mathrm{Fuel}\;({\dot{E}}_{F})$	$\mathrm{Exergy}\;\mathrm{of}\;\mathrm{Product}\;({\dot{E}}_{P})$	$\mathrm{Exergy}\;\mathrm{of}\;\mathrm{Loss}\;({\dot{E}}_{L})$
Evaporator (EVAP)	${\dot{E}}_{4}-{\dot{E}}_{1}$	${\dot{E}}_{6}-{\dot{E}}_{5}$	-
Compressor for refrigeration cycle (CM_R)	${\dot{W}}_{CM\_R}+{\dot{E}}_{1}^{T}$	${\dot{E}}_{2\_1}^{T}+{\dot{E}}_{2\_1}^{M}-{\dot{E}}_{1}^{M}$	-
Mixer (MIX)	-	-	-
Gas cooler (GC)	${\dot{E}}_{2}-{\dot{E}}_{3}$	${\dot{E}}_{8}-{\dot{E}}_{7}$	-
Throttling valve (TV)	${\dot{E}}_{3\_1}^{M}-{\dot{E}}_{4}^{M}+{\dot{E}}_{3\_1}^{T}$	${\dot{E}}_{4}^{T}$	-
Compressor for power cycle (CM_P)	${\dot{W}}_{CM\_P}$	${\dot{E}}_{9}-{\dot{E}}_{3\_2}$	-
Heater (HE)	${\dot{Q}}_{HE}\left(1-\frac{T_{0}}{T_{HS}}\right)$	${\dot{E}}_{10}-{\dot{E}}_{9}$	-
Expander (EX)	${\dot{E}}_{10}-{\dot{E}}_{2\_2}$	${\dot{W}}_{EX}$	-
Overall (only refrigeration)	${\dot{Q}}_{HE}\left(1-\frac{T_{0}}{T_{HS}}\right)$	${\dot{E}}_{6}-{\dot{E}}_{5}$	${\dot{E}}_{8}-{\dot{E}}_{7}$
Overall (refrigeration and heat)	${\dot{Q}}_{HE}\left(1-\frac{T_{0}}{T_{HS}}\right)$	${\dot{E}}_{6}-{\dot{E}}_{5}+{\dot{E}}_{8}-{\dot{E}}_{7}$	0
Overall (refrigeration and power)	${\dot{Q}}_{HE}\left(1-\frac{T_{0}}{T_{HS}}\right)$	${\dot{E}}_{6}-{\dot{E}}_{5}+\;{\dot{W}}_{net}$	${\dot{E}}_{8}-{\dot{E}}_{7}$
Overall (heat, refrigeration, and power)	${\dot{Q}}_{HE}\left(1-\frac{T_{0}}{T_{HS}}\right)$	${\dot{E}}_{6}-{\dot{E}}_{5}+\;{\dot{W}}_{net}$+ ${\dot{E}}_{8}-{\dot{E}}_{7}$	0

**Table 3:** Assumptions for economic analysis.

Variable	Nomenclature	Unit	Value
The economic lifetime of the power plant	*n*	a	20
Annual full load hours	*τ*	h a^−1^	8000
Effective interest rate	*i_eff_*	%	10
Average general inflation rate	$r_{FC}$ $,r_{OMC}$	%	2.5
Total Capital Investment	*TCI*	$	6.32 *PEC* [29]
Fuel cost at the beginning of the first year	$FC_{0}$	$	0
Operating and maintenance cost at the beginning of the first year	$OMC_{0}$	$	0.05 *TCI* n^−1^

**Table 4:** Assumptions for the cost estimation of printed circuit heat exchangers (PCHEs).

Item	Nomenclature	Value	Unit
Overall heat-transfer coefficient	*U*	500 [32]	W m^−^^2^K^−^^1^
The fraction of metal per m^3^ of the heat exchanger	$f_{m}$	0.564 [27]	m^3^ m^−^^3^
The density of stainless steel	$Density_{SS}$	7800	kg m^−^^3^
Cost of stainless steel per unit mass	$Cost\;per\;unit\;mass_{SS}$	50 [32]	$ kg^−^^1^

**Table 5:** The values used for computing the costs of the compressor for refrigeration cycle (CM_R) and evaporator (EVAP) [33].

**CM_R**					
$C_{B}$ ($)	$X_{B}\;\left(\mathrm{kW}\right)$	M	$f_{M}$	$f_{P}$	$f_{T}$
98,400	250	0.95 [29]	1	1.5	1
**EVAP**					
$C_{B}$ ($)	$X_{B}\;$(m^2^)	M	$f_{M}$	$f_{P}$	$f_{T}$
32,800	80	0.68	1	1.3	1

**Table 6:** Exergetic optimization results for each operation scenario.

	*T*_0_ = 35 °C		*T*_0_ = 45 °C	
	*T_EVAP_* = 5 °C	*T_EVAP_* = 15 °C	*T_EVAP_* = 5 °C	*T_EVAP_* = 15 °C
**Operating parameters**				
*T*_10_ (°C)	220	220	220	220
*p*_10_ (bar)	200	200	200	200
*T*_3_ (°C)	50	50	40	40
*p*_3_ (bar)	95	95	112	112
${\mathrm{PR}}_{P}$ (-)	2.11	2.11	1.79	1.79
${\mathrm{PR}}_{R}$ (-)	2.48	1.92	2.91	2.27
$m_{P}$(kg h^−1^)	4558.22	3267.43	10,289.66	8080.17
$m_{R}$ (kg h^−1^)	3396.58	3776.06	4535.40	5138.80
$m_{P}/m_{R}$ (-)	1.34	0.87	2.27	1.57
**Energetic results**				
$\;\eta_{P}$ (%)	10.92	10.92	8.56	8.56
*COP* (-)	2.56	3.57	1.59	2.03
$\;COP_{overall}$ (-)	0.28	0.39	0.14	0.17
**Exergetic results**				
$\varepsilon_{P}$ (%)	27.35	27.35	22.52	22.52
$\varepsilon_{R}$ (%)	17.07	11.14	16.10	13.14
$\varepsilon_{Overall}$ (%)	4.67	3.05	3.63	2.93
${\dot{E}}_{Heating}/{\dot{E}}_{Cooling}$ (-)	2.73	3.95	5.08	5.90

**Table 7:** Levelized cost for four scenarios.

Scenarios	*T*_0_ = 35 °C, *T_EVAP_* = 5 °C	*T*_0_ = 35 °C, *T_EVAP_* = 15 °C	*T*_0_ = 45 °C, *T_EVAP_* = 5 °C	*T*_0_ = 45 °C, *T_EVAP_* = 15 °C
Levelized Cost ($ year^−1^)	96,382	78,524	146,241	124,441

## Nomenclature

*A*	area [m^2^]
$\dot{C}$	cost rate associated with an exergy stream [$ (h)^−1^]
c	cost per unit of exergy [$ (GJ)^−1^]
*COP*	coefficient of performance [-]
$\dot{E}$	exergy rate [W]
*e*	specific exergy [kJ kg^−1^]
*f*	fraction of metal per m^3^ of the heat exchanger [m^3^ m^−3^]/correction factor [-]
*i*	interest rate [%]
*LMTD*	log-mean temperature difference [K]
$\dot{m}$	mass flow rate [kg s^−1^]
*n*	economic life of the plant [year]
*p*	pressure [bar]
$\dot{Q}$	heat rate [W]
*r*	inflation rate [%]
*T*	temperature [K, ºC]
*U*	overall heat-transfer coefficient [W m^−2^ K^−1^]
*V*	volume [m^3^]
$\dot{W}$	power [W]
$\dot{Z}$	cost rate [$ (h)^−1^]

## Greek Symbols

*ε*	exergy efficiency [%]
ρ	density [kg (m^3^)^−1^]
τ	annual operating hours [h (year)^−1^]
*η*	efficiency [-]

## Abbreviations

CC	carrying charges
CELF	constant escalation levelization factor
CM_R	compressor of the refrigeration cycle
CM_P	compressor of the power cycle
CRF	capital recovery factor
COM	component object model
EVAP	evaporator
EX	expander
FC	fuel cost
GA	genetic algorithm
GC	gas cooler
HE	heater
HS	heat source
MIX	mixer
OMC	operating and maintenance cost
ORC	organic Rankine cycle
PCHE	printed circuit heat exchanger
PEC	purchased equipment cost
PRc	compressor pressure ratio
SBC	supercritical Brayton cycle
SCO_2_	supercritical carbon dioxide
SPLIT	splitter
TCI	total capital investment
TCO_2_	transcritical carbon dioxide
TIP	expander (=turbine) inlet pressure
TIT	expander (=turbine) inlet temperature
TRR	total revenue requirement
TV	throttling valve
VCRC	vapor-compression refrigeration cycle

## Subscripts and Superscripts

*0*	reference state (dead state)/ the first year
*a*	average
*B*	base case
*CI*	capital investment
*Cooling*	refrigeration capacity
*D*	exergy destruction
*eff*	effective
*EVAP*	evaporator
*F*	exergy of fuel
*Heating*	heat capacity
*k*	*k*th component
*L*	levelized
*M*	mechanical
*P*	exergy of product/power cycle
*PH*	physical
*R*	refrigeration cycle
*T*	thermal/temperature
*tot, overall*	total
